# A Novel Technique for the Management of Blandin-Nuhn Mucocele: A Case Report

**DOI:** 10.5005/jp-journals-10005-1219

**Published:** 2013-10-14

**Authors:** Ramesh Kumaresan, Priyadarshini Karthikeyan, Faraz Mohammed, Arishiya Thapasum Fairozekhan

**Affiliations:** Lecturer, Department of Oral and Maxillofacial Surgery, AIMST University, Kedah, Malaysia; Postgraduate Student, Department of Oral Medicine and Radiology Sri Balaji Dental College, Chennai, Tamil Nadu, India, e-mail: priyadrkarthi@gmail.com; Assistant Professor, Department of Oral and Maxillofacial Pathology Dr. Syamala Reddy Dental College Hospital and Research Centre Bengaluru, Karnataka, India; Assistant Professor, Department of Oral Medicine and Radiology Dr. Syamala Reddy Dental College Hospital and Research Centre Bengaluru, Karnataka, India

**Keywords:** Mucocele, Glands of Blandin-Nuhn, Tongue

## Abstract

Mucocele, a common benign cystic lesion of minor salivary gland and associated ducts develops following extravasation or retention of mucous material in the subepithelial tissue. Occurrence of mucocele of tongue is considered less frequent when compared to a higher incidence of mucocele in the lower lip of young patients. Different modalities of treatment, such as conventional surgical excision followed by newer techniques like cryosurgery, electrocautery have been proposed to completely remove the lesion and reduce the chances of recurrence. Herewith, we report a novel treatment technique using alginate impression material to aid in complete excision of mucocele of glands of Blandin-Nuhn.

**How to cite this article:** Kumaresan R, Karthikeyan P, Mohammed F, Fairozekhan TA. A Novel Technique for the Management of Blandin-Nuhn Mucocele: A Case Report. Int J Clin Pediatr Dent 2013;6(3):201-204.

## INTRODUCTION

Mucoceles are cystic lesions found on any mucosal surfaces where underlying accessory salivary glands are present. They commonly appear as translucent bluish nodules that arise mostly on the lower labial mucosa followed by tongue, but other regions of oral cavity may also be affected.^1^Mucoceles involving the glands of Blandin-Nuhn (anterior lingual salivary glands), which are chiefly mucos-secreting glands that are embedded within the muscles of anterior tongue ventrum, are less frequent and constitute a small percentage of the reported cases, ranging from 1.9 to 10.3%.^2^As the glands of Blandin-Nuhn are not encapsulated and are directly overlapped to the muscle tissue, their manipulation tends to be different from the other oral mucoceles.^3^

Mucoceles of the glands of Blandin-Nuhn are exophytic and may resemble pyogenic granulomata, polyps or squamous papillomata. Clinically, a mucocele shows a positive history of trauma in most cases, rapid onset, alterations in size, bluish color and fluid-filled consistency.^2,4^The variation in color depends on size of lesion, its proximity to mucosal surface and elasticity.^5^

Treatment usually consists of surgical excision of the lesion including the involved minor salivary gland. While other techniques, such as cryosurgery and laser ablation were indicated for complete removal of the lesion.^6-8^

The present report discusses a case of mucocele from glands of Blandin-Nuhn treated by surgical excision, where an unexpected surgical difficulty was overcome.

## CASE REPORT

A 9-year-old boy was brought to our Oral Surgery Clinic by his mother with the chief complaint of swelling on the ventral aspect of the tongue of 1 month duration. The swelling had gradually increased in size and interfered with speech and mastication. There was no history of trauma to the area, and the medical history was not significant.

**Fig. 1:**
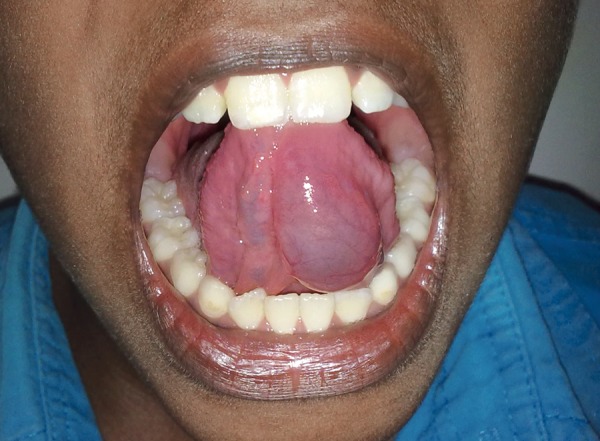
Clinical presentation of mucocele of the glands of Blandin-Nuhn

On clinical examination, a bluish, nonulcerated, oval shaped mass measuring about 25 × 15 mm in size was noticed on the left ventral aspect of the tongue. The swelling was painless, soft and mucosa over the lesion was tensed (Fig. 1). On aspiration, the swelling recovered mucos. The swelling was clinically diagnosed as a mucocele of glands of Blandin-Nuhn based on the peculiar location. Blood investigation revealed the parameters within the normal limits.

**Fig. 2:**
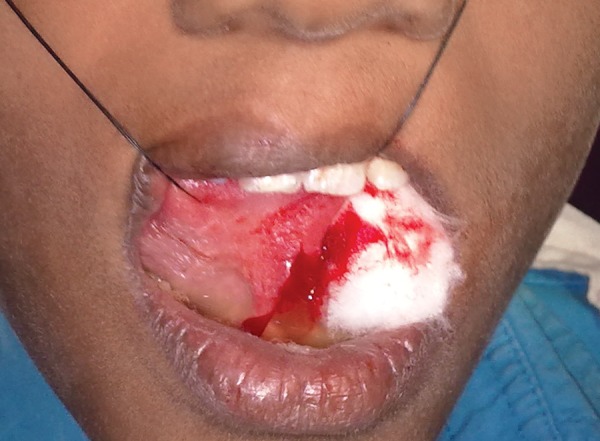
Extravasation of mucos during the surgical excision

Surgical removal of the lesion was planned under local anesthesia with 2% lignocaine. A vertical incision was made on the ventrum of the tongue and excision of the lesion was attempted. In course of dissecting the lesion from the underlying tissue, the cystic lining ruptured leading to an immediate extravasation of mucos (Fig. 2). This made the lesion to collapse causing difficulty in further surgical exploration.

**Fig. 3:**
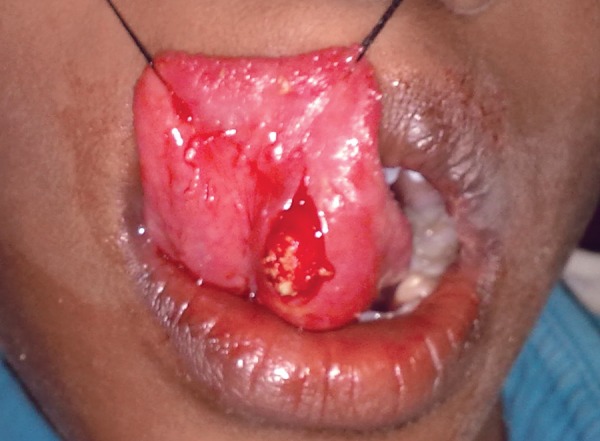
Lesion filled with alginate impression material

**Fig. 4:**
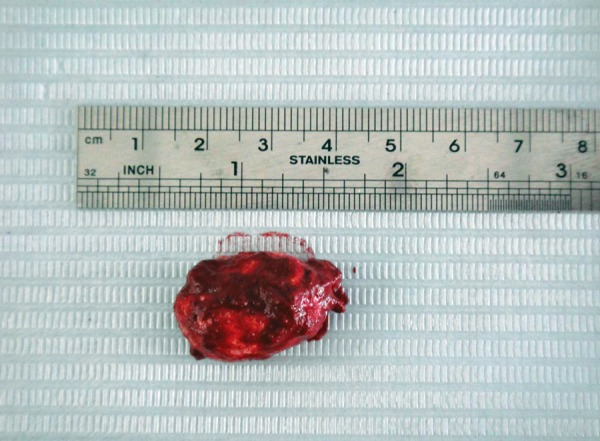
The gross appearance of the lesion after surgical removal

To improve the visual access of the lesion, a thin mix of alginate impression material was injected into the lesion through the ruptured wall using a 19 gauge needle. This technique helped in clearly demarcating the boundaries of the lesion (Fig. 3). The lesion was excised by dissecting down to the muscle layer and removed *in toto* together with the associated minor salivary glands (Fig. 4) to avoid recurrence. The wound was approximated with 4-0 sutures. The postoperative course was uneventful. The excised mass was sent for histopathological examination.

**Fig. 5:**
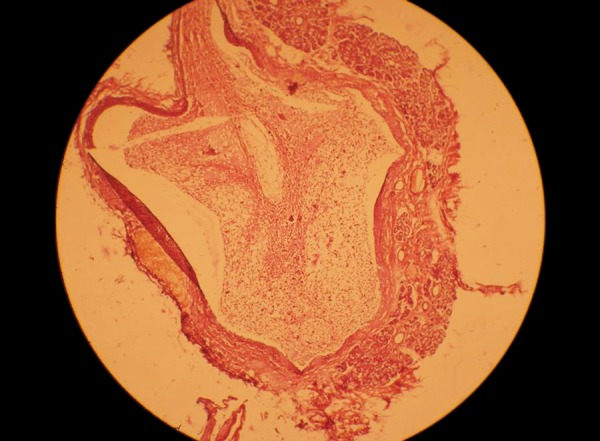
Histology of the lesion showing epithelial lining. Hematoxylin and Eosin stain. Magnification 4x

**Fig. 6:**
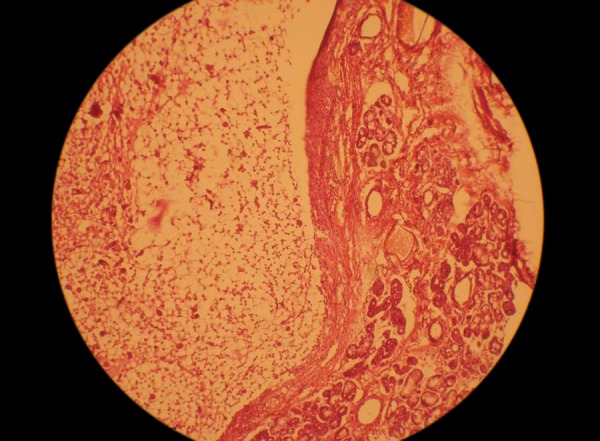
Histology of the lesion showing epithelial lining. Hematoxylin and Eosin stain. Magnification 10x

Histopathologic report revealed a Hematoxylin and Eosin stained section showing a well-delineated cavity containing eosinophilic mucinous material (Fig. 5). The cystic lining was composed of granulation tissue with fibroblasts, proliferating small caliber blood vessels and a mixed, acute and chronic inflammatory cell infiltration. At the periphery, few salivary glands were also evident (Fig. 6). The overall histopathological features were consistent with the clinical diagnosis of mucous extravasation phenomenon.

## DISCUSSION

Cystic swellings associated with minor salivary glands are referred to as mucocele or mucos cyst. Mucocele, (Muco – mucos & Coele – cavity) by definition, is a cavity filled with mucos. They commonly present as either the mucous extravasation type or mucos retention type. The former is the primary cause of mucocele formation involving exudation of mucos into the connective tissue and surrounded by a granulation tissue envelope. The mucos retention type on the other hand appears due to a decrease or absence of glandular secretion produced by blockage of the salivary gland duct.^9^ There is however, no clinical difference between extravasation and retention mucoceles.^5^Though, there appears to be no age and sex predilection, some studies state the lesion to appear mostly in the first three decades of life.^10^ Although, mucoceles can occur in any location where minor salivary glands are present, they are commonly found in the lower lip and less frequently reported on the tongue. However, few cases were reported on the ventral aspect of the tongue where the glands of Blandin and Nuhn are located.^11^

Diagnosis is principally clinical. The appearance of mucocele is pathognomonic and the following data are considered helpful in the clinical diagnosis: a history of trauma, rapid onset, variation in size, a fluid-filled consistency, bluish color and recovery of mucos with aspiration.^1,4,11-13^

Small mucoceles are treated by surgical excision which includes the servicing mucous glands with evacuation of its contents. On the other hand large lesions may be treated by cryosurgery,^6^ laser ablation^7^, micromarsupialization and steroid injection.^8^ Baurmash^5^ has advised on completely unroofing the lesion along its entire periphery to visualize and remove all of the glands present to prevent recurrence of the lesion.

During surgical excision on the ventral surface of the tongue, all the minor salivary gland tissues in the surgical field may be electively excised to avoid iatrogenic recurrence of a mucocele by residual salivary gland tissue left behind.^2^ In addition, incomplete excision of the cystic lining may also result in recurrence of the lesion. Hence, clearly demarcating the limit of the lesion is of prime importance in order to make the surgical excision complete and easier. Filling the cystic cavity with ultraflow rubber based impression material or alginate impression material presurgically improves the visual access for surgical excision.^14^ However, use of alginate is not recommended in areas where extension into surrounding tissue planes is possible leading to a foreign-body reaction in the tongue as a result of residual alginate within the tissue.^15^

In our case, during surgical exploration of the mucocele an unexpected rupturing of the lesion had occurred making the surgical exploration difficult. A thin mix of alginate impression material injected into the lesion clearly delineated the entire lesion and complete excision was made possible. No foreign-body reaction was noted post-operatively.

The use of alginate impression material to define the boundaries of the lesion is an effective, safe and economical way of treating an anterior lingual salivary gland mucocele, whilst ensuring complete removal and minimal chance of a recurrence. If performed correctly, this technique will undoubtedly prove to improve the surgical compliance thereby curtailing the recurrence of mucocele in the oral cavity.
